# Probing the Behaviour of Cas1-Cas2 upon Protospacer Binding in CRISPR-Cas Systems using Molecular Dynamics Simulations

**DOI:** 10.1038/s41598-019-39616-1

**Published:** 2019-02-28

**Authors:** Hua Wan, Jianming Li, Shan Chang, Shuoxin Lin, Yuanxin Tian, Xuhong Tian, Meihua Wang, Jianping Hu

**Affiliations:** 1 College of Mathematics and Informatics, South China Agricultural University, Guangzhou, 510642 China; 2 Institute of Bioinformatics and Medical Engineering, School of Electrical and Information Engineering, Jiangsu University of Technology, Changzhou, 213001 China; 3 Department of Electrical and Computer Engineering, James Clark School of Engineering, University of Maryland, College Park, MD 20742 USA; 4 School of Pharmaceutical Sciences, Southern Medical University, Guangzhou, 510515 China; 5 College of Pharmacy and Biological Engineering, Sichuan Industrial Institute of Antibiotics, Key Laboratory of Medicinal and Edible Plants Resources Development of Sichuan Education Department, Antibiotics Research and Re-evaluation Key Laboratory of Sichuan Province, Chengdu University, Chengdu, 610106 China

## Abstract

Adaptation in CRISPR-Cas systems enables the generation of an immunological memory to defend against invading viruses. This process is driven by foreign DNA spacer (termed protospacer) selection and integration mediated by Cas1-Cas2 protein. Recently, different states of Cas1-Cas2, in its free form and in complex with protospacer DNAs, were solved by X-ray crystallography. In this paper, molecular dynamics (MD) simulations are employed to study crystal structures of one free and two protospacer-bound Cas1-Cas2 complexes. The simulated results indicate that the protospacer binding markedly increases the system stability, in particular when the protospacer containing the PAM-complementary sequence. The hydrogen bond and binding free energy calculations explain that PAM recognition introduces more specific interactions to increase the cleavage activity of Cas1. By using principal component analysis (PCA) and intramolecular angle calculation, this study observes two dominant slow motions associated with the binding of Ca1-Cas2 to the protospacer and potential target DNAs respectively. The comparison of DNA structural deformation further implies a cooperative conformational change of Cas1-Cas2 and protospacer for the target DNA capture. We propose that this cooperativity is the intrinsic requirement of the CRISPR integration complex formation. This study provides some new insights into the understanding of CRISPR-Cas adaptation.

## Introduction

CRISPR-Cas (clustered regularly interspaced short palindromic repeat-CRISPR associated) systems, which have been identified in genomes of most archaea and almost half of the bacteria, confer resistance to viral infection by detecting and cleaving invading nucleic acids^1–3^. The CRISPR-Cas system establishes the adaptive immunity in three stages: adaptation, expression and interference^4,5^. In the adaptation step, the fragment of foreign DNA (termed protospacer) is selected and inserted into the CRISPR array as a new spacer^6,7^. Through spacer integration, the captured spacer sequence serves as the genetic memory of viral infections to recognize and defend against the further attacks from the same virus. Hence, uncovering the adaptation process is essential for understanding the whole machinery of CRISPR-Cas defense.

In recent years, a series of studies focused on investigating molecular mechanism of CRISPR-Cas adaptation^6,8–17^^.^ The protospacer-adjacent motifs (PAMs) locate in the protospacer flanking region, and contain 2 to 7 nucleotides (nt)^8,9^. Mutations in the PAM can abolish CRISPR-mediated immunity in *Escherichia coli*^9,10^. Through spacer composition analysis, it was found that the last nucleotide of the new repeat is PAM derived^11^. Besides, Cas1 and Cas2 are two highly conserved Cas proteins in almost all CRISPR-Cas systems^12^. Overexpression of Cas1 and Cas2 was shown to be sufficient for new spacer acquisition^13^. To further reveal the structural basis for spacer acquisition, the group led by Doudna determined the crystal structure of Cas1-Cas2 complex at 2.3 Å resolution in 2014^6^. The overall structure contains a central Cas2 dimer and a pair of flanking Cas1 dimers. In 2015, Doudna *et al*. continued to publish two structures of the Cas1-Cas2-protospacer complex in the presence and absence of Mg^2+^ at 3.0 Å and 3.2 Å resolutions, respectively^14^. Separately, another group led by Wang solved the crystal structures of Cas1-Cas2 with a series of protospacer substrate DNAs at 2.6~4.5 Å resolutions^15^. The two groups both described that the protospacer lies across the flat surface formed by Cas1-Cas2, with each single-stranded 3′ end binding at the Cas1 active site. The electrophoretic mobility shift assay (EMSA) analysis showed that the spacer acquisition is PAM-dependent^15^. A pair of new studies^16,17^ in 2017 reported the half and full integration structures of Cas1-Cas2-protospacer-target, and revealed that the bending of target DNA is important for the recognition and integration of spacer-side active site. These important structure data provided valuable clues for studying the spacer acquisition machinery in CRISPR-Cas systems.

In addition to experiments, theoretical methods^18–23^ were also adopted to explore the classification, evolution, structure and function of CRISPR-Cas systems. Multiple sequence alignments and hidden Markov models were built for the identification of new Cas protein families^18^. Martynov *et al*. theoretically estimated the number of spacers in CRISPR arrays that maximizes its protection against a viral attack^19^. Pinello *et al*. developed a suite of computational tools CRISPResso to evaluate the outcomes of CRISPR genome editing experiments^20^. The coarse-grained modeling was utilized to study the dynamics of CRISPR-Cas9 interacting with DNA and RNA^21^. Molecular dynamics (MD) simulations were performed to probe the process of R-loop (DNA-RNA) formation in CRISPR-Cas9 system^22^. Cas9 DNA cleavage specificity correlates with the stability of R-loop complex structures based on a statistical mechanical analysis^23^. These theoretical works enhance our understanding of the adaptive immunity mechanism of CRISPR-Cas system. Nevertheless, the crystal structures of Cas1-Cas2 bound to protospacer DNA, serving as the essential adaptation elements, have not been simulated systemically. The experimental and crystallographic studies^6,11,15^ showed that in *E*. *coli* the spacer acquisition is associated with PAM recognition. The reverse side of flat surface provided by Cas1-Cas2 is involved in the recognition of the target DNA^16,17^. The protospacer DNA binding induces a significant conformational change of Cas1-Cas2^14,15^, in the meantime, bending deformations of both protospacer and target DNAs were observed^15–17^. Then, several important questions still need to be answered. What atomic level interactions does PAM recognition contribute to the protein-DNA binding? How do the Cas1-Cas2 dynamics affect the binding to both protospacer and target DNAs? What DNA deformations occur at base pair (bp) level? Are there any underlying binding mechanism to explain the conformational change of Cas1-Cas2 and DNA?

To address the above issues, the three crystal structures of one DNA-free and two protospacer DNA-bound Cas1-Cas2 complexes were studied by MD simulations. The principal component analysis and free energy landscape methods were used to investigate the slow motions of Cas1-Cas2 and protospacer DNA. We defined two intramolecular angles to measure the dominant conformational changes. To explain differences of the distributions in conformational space between DNA-bound systems, the crucial interactions were examined at the protein-DNA interfaces by the hydrogen bond and binding free energy calculations. Then, we observed different hydrogen-bonding patterns in the two DNA-bound structures. Additionally, the Cas1-Cas2-binding-induced DNA deformations were analyzed at bp level. By exploring the correlation between the open-close conformational change of Cas1-Cas2 and the localized bending of DNA, we proposed a cooperative binding between Cas1-Cas2 and protospacer for the target DNA capture.

## Systems and Methods

### Structures of free and protospacer-bound Cas1-Cas2

**Figure 1:**
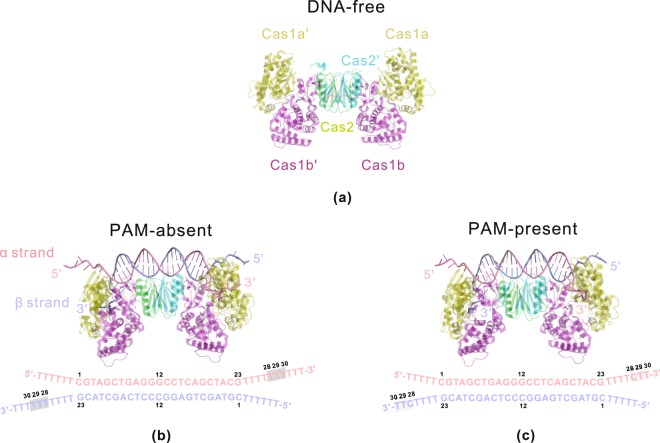
Overall architectures of DNA-free and DNA-bound Cas1-Cas2. (**a**) Structure of Cas1-Cas2 in its free form. Cas1a/Cas1a′ (yellow) and Cas1b/Cas1b′ (magenta) sandwich Cas2 (green) and Cas2′ (cyan). (**b**,**c**) Two structures of Cas1-Cas2 bound to protospacer DNA (α strand: pink; β strand: light blue), having the substantial difference at nucleotides 28~30. The nucleotide segment T28-T29-T30 is highlighted by gray background (**b**), and C28-T29-T30 (termed PAM-complementary sequence) is highlighted by light gray background (**c**).

The three crystal structures of *E*. *coli*, including free Cas1-Cas2 (PDB code: 4P6I), Cas1-Cas2 bound to protospacer DNA with PAM-complementary sequence absent (PDB code: 5DLJ) and with PAM-complementary sequence present (PDB code: 5DQZ), were obtained from the Protein Data Bank^15^. As described by Fig. 1a, Cas1-Cas2 contains four Cas1 and two Cas2 subunits, showing an overall architecture of one Cas2 dimer (labeled Cas2/Cas2′) being sandwiched between two Cas1 dimers (labeled Cas1a/Cas1b and Cas1a′/Cas1b′). The two DNA-bound Cas1-Cas2 structures are highly similar (Fig. 1b,c). The protospacer sequence consists of a 23-bp duplex flanked by short overhangs. The duplex segment binds to the flat surface provided by Cas1-Cas2, with the two 3′ overhangs threading into the C-terminal domain of Cas1a and Cas1a′. There is only one nucleotide difference at position 28 between the two DNA-bound structures, which leads to two different types of nucleotide segments at positions 28~30 (Fig. 1b,c, labeled by gray and light gray backgrounds). The type of nucleotide segment 5′-CTT-3′ is complementary to the PAM sequence 5′-AAG-3′ that was specifically recognized by the binding pocket residues within Cas1 and then was considered to be important for protospacer selection during acquisition^11,24^. For convenience, the free and two protospacer-bound Cas1-Cas2 systems were referred to as the DNA-free, PAM-absent and PAM-present systems, respectively.

### Simulation protocols

The three independent MD simulations were performed using the NAMD 2.9 software^25^ with CHARMM27 all-atom additive force field for nucleic acids^26^. The initial models were constructed by VMD 1.9^27^. Each structure was solvated with TIP3P water in a cubic periodic box, with the distance of ~10 Å between the solute unit and the edge of the box. Counter ions (Na^+^, Cl^−^) were added to neutralize the systems. Then, the three (DNA-free, PAM-absent and PAM-present) systems contained 206831, 284384 and 279971 atoms, respectively. For each system, a two-stage simulation was carried out. Firstly, the system was energetically minimized with 50000 steps and then slowly heated over 1 ns from 0 K to 310 K. The positions of Cas1-Cas2 protein and protospacer DNA were restrained with a harmonic constant of 0.1 kcal·mol^−1^ Å^−2^ to avoid improper geometry. After that, the non-restraint MD simulation was run at constant pressure (1 atm) by the Langevin piston method^28^ for 100 ns. In these simulations, bonds involving hydrogen atoms were fixed with the SHAKE algorithm^29^. Electrostatic interactions were evaluated using the particle mesh Ewald (PME) method^30^. The cut-off value for non-bonded interactions was set to 12 Å. The MD trajectory snapshots were saved every 2.0 ps and thus 50000 conformations were collected for further analysis.

### Principal component analysis

Principal component analysis (PCA) is an effective method for analyzing trajectory data from MD simulations to find the essential dynamics^31,32^. This method uses a linear transformation to project the original high-dimensional representation of biomacromolecular dynamics into the low-dimensional space based on the calculation of covariance matrix. The elements of the covariance matrix *C*_*ij*_ are represented as^33^:

1 $${C}_{ij}=\langle ({x}_{i}-\langle {x}_{i}\rangle )({x}_{j}-\langle {x}_{j}\rangle )\rangle $$

where *x*_*i*_ (*x*_*j*_) is the coordinate of the *i* th(*j* th) atom and <⋯> denotes an ensemble average. The matrix is diagonalized to get the set of eigenvectors (i.e., principal components) and eigenvalues, which determine the directions of the concerted motions and the magnitudes of the motions along the directions. Then, principal components (PCs) are sorted in terms of the eigenvalue contribution to the whole atomic movement. Because the large-scale motions are often the most biologically relevant, the first few PCs are required to capture a large portion of the variance of atom positional fluctuations when they are selected to describe the essential dynamics of biomacromolecules.

In practice, it is also important to assess the robustness of the PCA modes by checking the cosine content (*cc*_*i*_) of PCs^34,35^. *cc*_*i*_ is given by^35^:

2 $$c{c}_{i}=\frac{2}{T}{({\int }_{0}^{T}\cos (i\pi t){p}_{i}(t)dt)}^{2}{({\int }_{0}^{T}{p}_{i}^{2}(t)dt)}^{-1}$$

where *p*_*i*_(*t*) is the amplitude of the motion along eigenvector *i* at time *t*. *cc*_*i*_ varies from 0 to 1. If MD trajectories could not provide sufficient sampling of conformational space, the first few PCs will resemble cosine functions which occur for a random diffusion process. Hence, the value of *cc*_*i*_ being close to 1 means limited conformational sampling. In this article, PCA was performed with GROMACS 5.1 package^36^ to investigate the dominant motions of protospacer DNA-bound systems.

### Free energy landscape

Free energy landscape (FEL) provides a useful description on conformation exchange during the biomolecular processes, such as molecular recognition, folding and aggregation^37^. Free energy basins and their depths determine the population and stability of functionally distinct states while the inter-basin barriers correspond to the transient states connecting them. The relative free energy between two states is specified by:

3 $${G}_{1}(X)-{G}_{2}(X)=-\,{K}_{B}T\,\mathrm{ln}[\frac{{P}_{1}(X)}{{P}_{2}(X)}]$$

where *K*_*B*_ is the Boltzmann constant, *T* is absolute temperature, *X* stands for the reaction coordinate and *P*(*X*) is the probability distribution of the system along the reaction coordinate. In this study, PC1 and PC2 were chosen as reaction coordinates based on the above PCA calculation. Then, two-dimensional free energy landscapes were constructed to analyze the effect of PAM recognition on conformational distributions of protospacer DNA-bound systems.

### Analysis of the interfacial interactions

To identify the critical contacts at residue-base level, the interfacial interaction analysis was done by the hydrogen bond and binding free energy calculations in this article. The hydrogen bond calculation was performed by VMD 1.9^27^ with a distance cut-off value of 3.5 Å and an angle cut-off value of 45°. Molecular Mechanics/Generalized Born Surface Area (MM/GBSA) and Molecular Mechanics/Poisson-Boltzmann Surface Area (MM/PBSA) methods are both popular approaches to analyze binding free energies between protein and ligand^38–41^. By using g_mmpbsa tool of GROMACS^42^, we obtained various MM/PBSA terms including molecular mechanics potential energy (electrostatic and van der Waals energies), free energy of solvation (polar and nonpolar solvation energies) as well as the energetic contribution of each residue and nucleotide to total binding energy.

### Conformational analysis of nucleic acids

The Curves program^43^ is widely used in nucleic acid conformational analysis by providing a full set of DNA structural parameters at bp level. In this study, 5000 snapshots were extracted from the equilibrium trajectories of the two protospacer DNA-bound systems by sampling every 10 ps. The Cas1-Cas2-binding-induced deformations of DNA were evaluated via groove width and bp-axis bend parameters.

## Results and Discussion

### MD results

**Figure 2:**
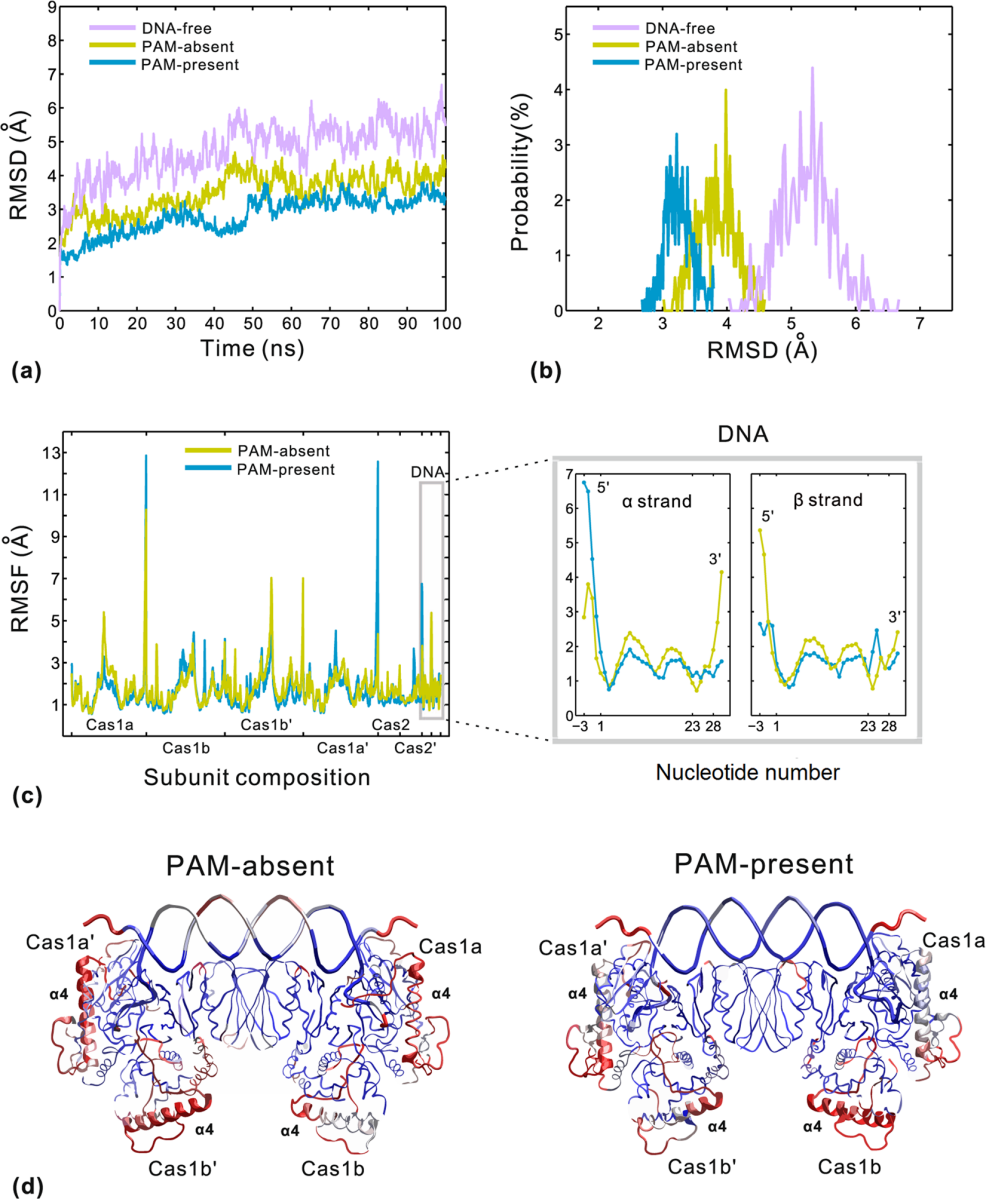
Comparative MD analyses of the DNA-free (purple), PAM-absent (yellow) and PAM-present (blue) systems. (**a**) The RMSDs of backbone atoms versus simulation time. (**b**) The probability distribution of RMSDs calculated from the equilibrium trajectories. (**c**) The RMSFs of the PAM-absent and PAM-present systems calculated from the equilibrium trajectories (left), and a magnified RMSFs view of DNA for clarity (right). (**d**) The representations of common regions of PAM-absent (left) and PAM-present (right) systems. The residues in red have relatively higher RMSF values (>2.5 Å) while the ones in blue have relatively lower RMSF values (<1.5 Å). The other regions are colored white.

Three 100 ns MD simulations were carried out for the DNA-free, PAM-absent and PAM-present systems, respectively. Figure 2a shows the root mean square deviation values (RMSDs) of backbone atoms in the three systems. The last 50 ns MD trajectories keep comparatively stable and are taken as the equilibrium portions. Figure 2b compares the distributional probability of RMSDs from the equilibrium trajectories in the three systems. The RMSDs converge to about 5.2 Å, 3.8 Å and 3.2 Å for the DNA-free, PAM-absent and PAM-present systems, respectively. The RMSDs of the two DNA-bound systems are significantly lower than those of the DNA-free system, indicating that the protospacer DNA binding has a dramatic effect on the protein stability. In the two DNA-bound systems, the PAM-present system is relatively more stable than the PAM-absent system. The substantial difference between them is that the 3 overhangs have different types of nucleotide segments at positions 28~30 (Fig. 1b,c). Thus, the PAM-complementary sequence (C28-T29-T30) is suggested to be primarily responsible for the increased stability of the PAM-present system. Our simulated result is consistent with the importance of PAM-complementary sequence in the Cas1-Cas2-protospacer binding by EMSA analysis^15^.

To further investigate the effect on stability made by the PAM-complementary sequence, we compare the dynamic fluctuation at residue level by calculating the root mean square fluctuation values (RMSFs) of the two DNA-bound systems. The RMSF calculation was performed for their common regions composed of 1250 Cα and 68 P atoms. As shown in Fig. 2c, the two DNA-bound systems have similar RMSF distributions and the correlation coefficient is 0.74. In order to intuitively compare the fluctuations, the two structures are colored according to the RMSFs (Fig. 2d). The regions with low and high RMSFs are shown in blue and red, respectively. In both systems, the red regions are mainly located on helix α4 (residues 134~156) and two adjacent loops of α4 (residues 124~133 and 157~168) in Cas1 dimers. Obviously, helix α4 undergoes remarkable motions in response to large conformational changes. Additionally, there are some noticeable differences of RMSFs between the two systems. At the 3′ end of DNA, positions 28~30 of the PAM-present system have lower fluctuations than those of the PAM-absent system, particularly remarkable in α strand (Fig. 2c right). It demonstrates that the PAM-complementary sequence of the PAM-present system is better constrained by the catalytic residues in Cas1 subunits. Meanwhile, the duplex segment (positions 1~23) of the PAM-present system also shows relatively lower RMSFs in comparison with that of the PAM-absent system. Then, we speculated that the improved protein-DNA binding contributed by the PAM-complementary sequence not only directly stabilizes the 3′ end of DNA, but also helps the reduction in the fluctuations of duplex segment of DNA to some extent (Fig. 2d). The comparative analyses of MD trajectories suggest that the PAM-complementary sequence functions as the PAM sequence in improving the performance of protospacer selection during spacer acquisition^8,9^.

### Functional conformational changes

**Figure 3:**
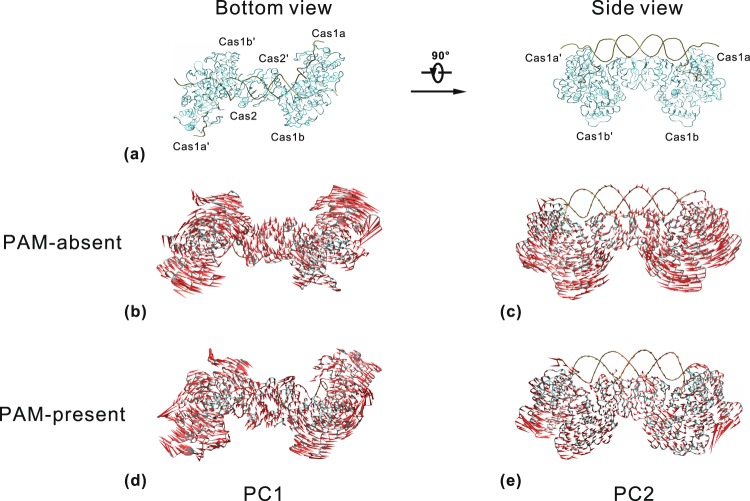
The slowest motion modes of the PAM-absent and PAM-present systems. (**a**) The bottom view (left) and side view (right) of the structure of Cas1-Cas2-DNA. (**b**,**d**) The first slowest motion mode using the bottom view (**a** left). (**c**,**e**) The second slowest motion mode using the side view (**a** right). The length of cone is positively-correlated with motive magnitude, and the orientation of cone indicates motive direction.

The similar fluctuation distributions revealed by the RMSFs analysis imply that the two DNA-bound systems may have similar conformational transitions. Then, the PCA analysis was performed to inspect the directions of motions based on the equilibrium trajectories. To evaluate the robustness of PCA results, we checked the cosine contents of the first eight PCs. The cosine content values of PC1~PC8 of the two DNA-bound systems are listed in Supplementary Table S1. The previous studies^44,45^ suggested that the threshold value of cosine content might be 0.2 for small peptides and 0.5 for proteins to discriminate sufficient sampling. In our study the cosine content values are relatively low (<= 0.3850), indicating that these movements corresponding to PCs are the genuine motions of Cas1-Cas2 protein and protospacer DNA. Supplementary Fig. S1 gives the proportion of variance of atom positional fluctuations of the first 50 PCs. The proportion rapidly decreases and converges to zero with the increasing of PC index in each system. The first two PCs together cover approximately 54.06% and 47.32% of total variance in the PAM-absent and PAM-present systems, respectively. Thus, PC1 and PC2 capture a higher fraction of the system’s variance in each system^46^. Then, we further compare the first and second slow motion modes of the two systems (Fig. 3). In each system, PC1 mainly exhibits the open-close movements on the surfaces of Cas1a-Cas2′-Cas1b′ and Cas1a′-Cas2-Cas1b (Fig. 3b,d) and PC2 appears as the rotation motions in reverse direction between the two Cas1 dimers (Fig. 3c,e). The high similarity of slow motion modes between the two systems suggests that these slow motions probably are associated with Cas1-Cas2 function.

**Figure 4:**
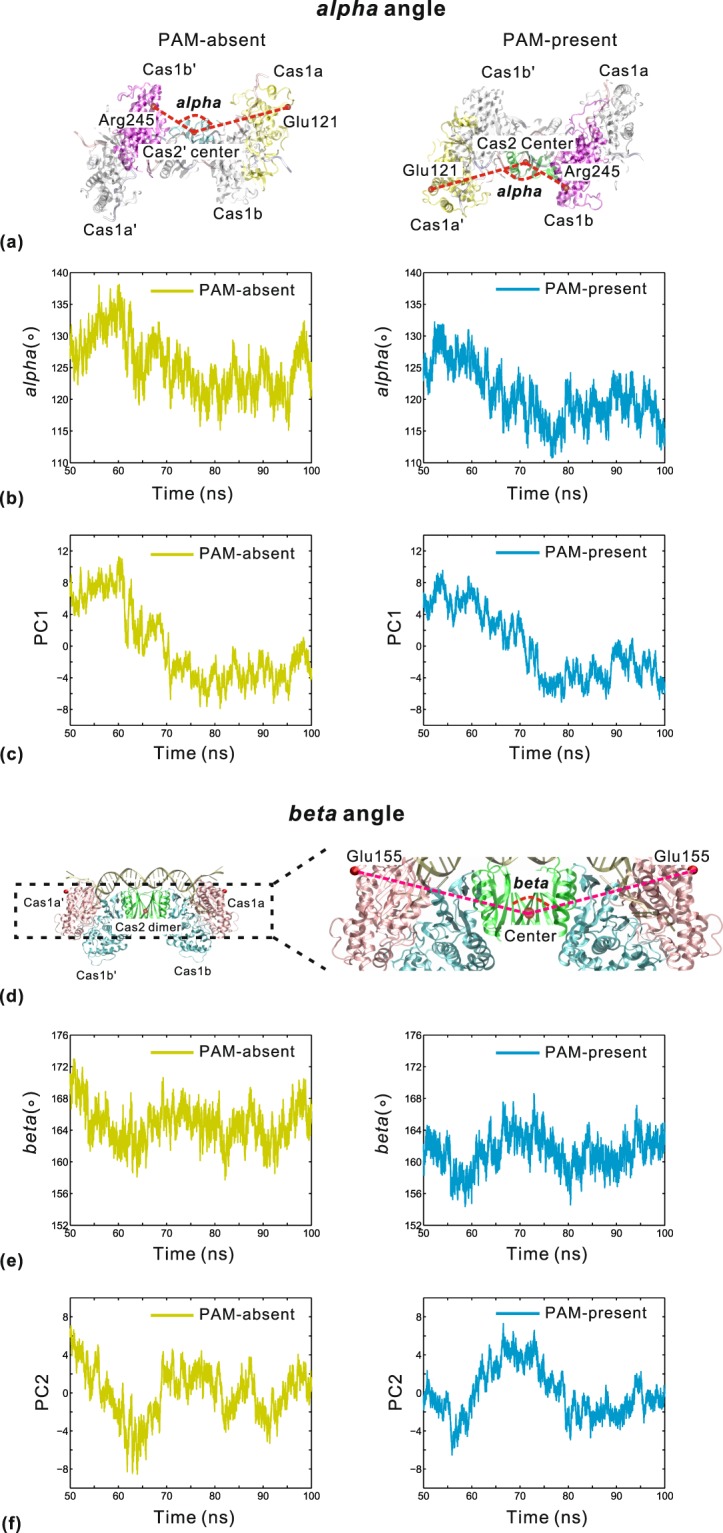
Comparisons of the intramolecular angle *alpha* (**a**,**b**) and *beta* (**d**,**e**) with PC1 (**c**) and PC2 (**f**), respectively. (**a**) The representation of *alpha* in the PAM-absent (left) and PAM-present (right) systems. It is defined by the Cα atom of Glu121 in Cas1a/Cas1a′ (yellow), the geometric center of Cas2′/Cas2 (cyan/green) and the Cα atom of Arg245 in Cas1b′/Cas1b (magenta). (**b**,**c**) Changes of *alpha* (**b**) and PC1 (**c**) versus simulation time. (**d**) The representation of *beta*. It is defined by the Cα atom of Glu155 of Cas1a′ (pink), the geometric center of Cas2 dimer (green) and the Cα atom of Glu155 of Cas1a (pink). (**e**,**f**) Changes of *beta* (**e**) and PC2 (**f**) versus simulation time.

According to the direction of the first slowest motion (Fig. 3b,d), we define an intramolecular angle *alpha* on the surfaces of Cas1a-Cas2′-Cas1b′ and Cas1a′-Cas2-Cas1b in the PAM-absent and PAM-present systems, respectively. The *alpha* angle is measured by the three points: the Cα atom of Glu121 of Cas1a/Cas1a′, the geometric center of Cas2′/Cas2 and the Cα atom of Arg245 of Cas1b′/Cas1b (Fig. 4a). The changes of *alpha* and PC1 versus simulation time are shown in Fig. 4b,c, respectively. In each system, the fluctuations of *alpha* values show a positive correlation with the changes of PC1 versus simulation time. It indicates that the open-close conformational change of PC1 can be well described by the *alpha* angle. In the two DNA-bound structures, Cas1-Cas2 always adopts a conformation in which the Cas2 dimer is positioned adjacent to Cas1b/Cas1b′ but far apart from Cas1a/Cas1a′. No contacts were observed between Cas1a/Cas1a′ and the Cas2 dimer^15^. The lack of enough constraint leads to a strong motility of Cas1a/Cas1a′ relative to the Cas2 dimer, which corresponds to these remarkable open-close movements of PC1. The most recently reported the full-integration structure of Cas1-Cas2-protospacer-target in 2017 (PDB code: 5XVP)^17^ revealed that the target DNA binds across the surfaces of Cas1a′-Cas2-Cas1b and Cas1a-Cas2′-Cas1b′. Then, we calculated the *alpha* angle from the DNA-free, PAM-absent, PAM-present and 2017 full-integration structures. The *alpha* angle values of the four structures are about 135.0°, 125.2°, 123.9° and 115.4°, respectively. The comparison reveals that the *alpha* angle is decreased for all DNA-bound structures, with the lowest value in 2017 full-integration structure. It is presumably because the target DNA introduces the additional protein-DNA contacts on the surfaces of Cas1a′-Cas2-Cas1b and Cas1a-Cas2′-Cas1b′. Hence, these open-close movements of the first slow motion are required by the process of Cas1-Cas2-protospacer binding to the target DNA, in which the surfaces of Cas1a′-Cas2-Cas1b and Cas1a-Cas2′-Cas1b′ intrinsically transit from open to closed conformation for accommodating the new sequence. After that, the target-bound structure will be expected to achieve higher stability.

The crystallographic studies^14,15^ showed that Cas1-Cas2 undergoes a remarkable conformational change upon protospacer DNA binding, with the two Cas1 dimers rotating in either clockwise or anti-clockwise directions. In our study, the second slowest motion mode also shows this rotation motion (Fig. 3c,e). To evaluate this conformational change, the second intramolecular angle *beta* is defined by the three points: the Cα atom of Glu155 in Cas1a′, the geometric center of the Cas2 dimer and the Cα atom of Glu155 in Cas1a (Fig. 4d). The changes of *beta* and PC2 versus simulation time are shown in Fig. 4e,f, respectively. The fluctuations of *beta* values are similar to those of PC2 versus simulation time in the two systems. Then, this rotation conformational change can be characterized by the angle *beta*. The *beta* angle values are 132.7°, 159.6°, 158.6° and 160.9° from the DNA-free, PAM-absent, PAM-present and 2017 full-integration structures (PDB code: 5XVP)^17^, respectively. Compared with the DNA-free structure, all the DNA-bound structures adopt a conformation with an increased *beta* angle. On the one hand, this conformational change provides a more flat protein surface favoring the association of the protospacer sequence. On the other hand, this conformational change corresponds to a structural rearrangement of Cas1 dimers and leads to the formation of the binding pocket within Cas1 subunits^15^. Altogether, these rotation motions of Cas1 dimers are necessary for the binding between Cas1-Cas2 and the protospacer sequence.

**Figure 5:**
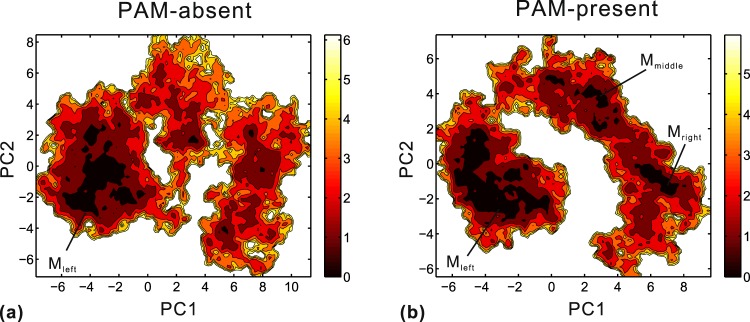
Free energy contour maps versus PC1 and PC2 for the PAM-absent (**a**) and PAM-present (**b**) systems. Deeper color corresponds to lower energy. M_left_, M_middle_ and M_right_ all stand for the local basins.

Additionally, we also investigated the conformational distributions along PC1 and PC2 based on equilibrium trajectories. Figure 5a,b display the free energy contour maps of the PAM-absent and PAM-present systems, respectively. The deeper color indicates lower energy. By comparison, there are some differences of the distributions in conformational space between the two DNA-bound systems. Firstly, the PC1 and PC2 of FEL_PAM-present_ span narrower ranges than those of FEL_PAM-absent_, revealing that the PAM-present system adopts conformational states narrower than those of the PAM-absent system. It is in good agreement with the RMSF analysis that the Cas1-Cas2 protein is better constrained in the PAM-present system. Second, FEL_PAM-present_ has three local basins (M_left_, M_middle_ and M_right_) while there is only one local basin (M_left_) in FEL_PAM-absent_. Thirdly, although M_left_ is the biggest local basin in each system, the low-energy conformations of the PAM-present system are still more populous than those of the PAM-absent system. To sum up, the PAM-present system achieves stable conformational states more efficiently than the PAM-absent system. In consideration of the structural difference between the two DNA-bound structures, we speculate that this different distribution should be related to different protein-DNA interaction mode involving PAM recognition. So, we analyze the interfacial interaction at residue-base level below.

### Protein-DNA Interactions

**Figure 6:**
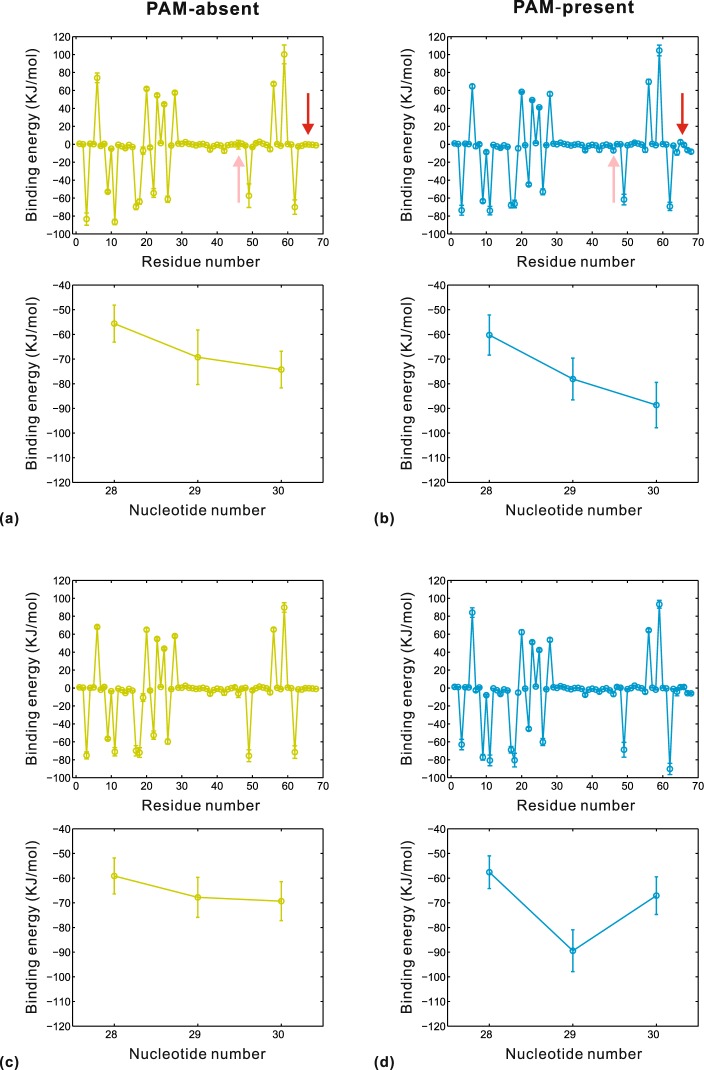
Binding energy decompositions at the protein-DNA interface between the binding pocket of Cas1a-Cas1b and α strand of DNA (**a**,**b**), and the protein-DNA interface between the binding pocket of Cas1a′-Cas1b′ and β strand of DNA (**c**,**d**). In each subfigure, the upper plot shows energetic contribution of residues and the lower plot shows energetic contribution of nucleotides. The pink and red arrows mark the positions of His208 in Cas1a and Gln287~Ile291 in Cas1b, respectively. Energies are given as kilojoules per mole. Error bars stand for the standard deviations of the energies.

In order to estimate the importance of PAM recognition, we calculated the interaction free energies between the binding pocket residues of Cas1 protein and the nucleotide segment at positions 28~30 of DNA. The detailed data is shown in Supplementary Table S2. Relative to the PAM-absent system, the PAM-present system has lower binding energies at both Cas1a-Cas1b-DNA and Cas1a′-Cas1b′-DNA interfaces. Specially, the van der Waals energy, electrostatic energy and nonpolar solvation energy of the PAM-present system are lower than those of the PAM-absent system, with the exception of the polar solvation energy. Because DNA is a polar molecule and the polar surface area of cytosine is larger than that of thymine^47^, C28-T29-T30 of the PAM-present system has more polarity than T28-T29-T30 of the PAM-absent system. This may cause high polar solvation energies in the two DNA-bound systems and this unfavorable effect is relatively stronger in the PAM-present system. Nevertheless, the total binding energy of the PAM-present system is lower which suggests that the PAM-complementary sequence (C28-T29-T30) forms more protein-DNA interactions. Then, the energy decomposition strategy was further used to investigate per-residue and per-nucleotide energetic contribution (Fig. 6). The two systems present similar energy distributions for binding pocket residues, with some small differences from His208 of Cas1a and Gln287~Ile291 of Cas1b (marked by pink and red arrows). These residues have favorable negative binding energies in the PAM-present system but this favorable contribution is lost in the PAM-absent system. Meanwhile, C28-T29-T30 of the PAM-present system has lower binding energies than T28-T29-T30 of the PAM-absent system. In particular, T29 of C28-T29-T30 always make a more favorable energetic contribution to Cas1 binding in comparison with T29 of T28-T29-T30 at both Cas1a-Cas1b-DNA and Cas1a′-Cas1b′-DNA interfaces. Then, we speculate that T29 of the PAM-complementary sequence participates in some interaction which is important for PAM recognition by Cas1.

**Figure 7:**
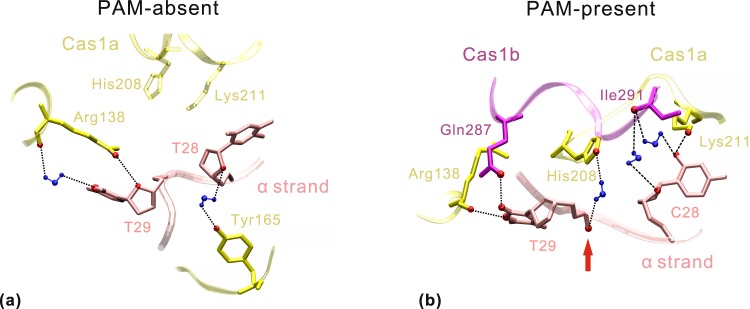
Different hydrogen-bonding patterns involving PAM recognition in the PAM-absent (**a**) and PAM-present (**b**) systems. The Cas1a/Cas1b (yellow/magenta), DNA (pink) and water (blue) are depicted with ribbon, tube and CPK models, respectively. Atoms involved in protein-DNA interactions are shown in CPK. The suggested DNA cleavage site at step C28-T29 is labeled by a red arrow (**b**).

Next, we examined the hydrogen bonds of the DNA-bound two systems at atomic level. The direct and water-mediated hydrogen bonds involving PAM recognition with occupancy over 20% are listed in Table 1. Consistent with the above analysis of binding energies, the PAM-complementary sequence forms more hydrogen bonds with Cas1 protein. Figure 7 describes the substantial difference of protein-DNA interactions involving nucleotides 28~29 at Cas1a-Cas1b-DNA interface. In the PAM-absent system (Fig. 7a), atom O4′ of T29 forms a direct hydrogen bond with NH1 of Tyr138 in Cas1a. Meanwhile, O4′ of T28 and N3 of T29 also form a water-mediated hydrogen bond with Tyr165 OH and Tyr138 O in Cas1a, respectively. In this case, His208 side chain in Cas1a keeps away from T29. In the PAM-present system (Fig. 7b), C28 adopts a different conformation in which its side chain forms one direct and two water-mediated hydrogen bonds with Lys211 in Cas1a and Ile291 in Cas1b, respectively. Meanwhile, N3 and O2 of T29 directly contact with O of Arg138 in Cas1a and NE2 of Gln287 in Cas1b, respectively. In this case, His208 in Cas1a donates a water-mediated hydrogen bond with the phosphate oxygen atom of T29. Notably, this hydrogen bond was considered to be essential for obtaining the cleavage site at C28-T29 step within 3′ overhangs through a cleavage assay^15^. However, this crucial interaction (Table 1, hydrogen bonds in bold) is weakened remarkably in the PAM-absent system. In the previous *in vivo* assay, the spacer acquisition was found to be reduced in different degrees on replacement of individual His208, Lys211 and Gln287 by alanine^6,15^. Our analysis of the interfacial interactions proves that these additional contacts in the PAM-present system improve the binding specificity between Cas1 and nucleotides C28-T29, which will increase the cleavage activity at C28-T29 step. Therefore, the PAM-complementary sequence plays a key role in improving the recognition efficiency of the 3′ overhangs by Cas1.

**Table 1:** The direct and water-mediated hydrogen bonds involving PAM recognition with occupancy over 20%.

Cas1	PAM-absent system			PAM-present system		
Subunit	Protein	DNA	HDO^※^	Protein	DNA	HDO^※^
Cas1a	*Tyr165-OH*	*T28- O4*′^*α*^	*42*.*56%*	Lys211-NZ	C28-O2^α^	30.21%
	Arg138-NH1	T29- O4′^α^	26.49%	Arg138-O	T29-N3^α^	73.91%
	*Arg138-O*	*T29- N3* ^*α*^	*24*.*68%*	***His208-NE2***	***T29-O1P*** ^**α**^	***20***.***56%***
	Tyr165-N	T30-O2^α^	46.09%			
	Tyr165-O	T30-N3^α^	41.89%			
	*Tyr165-OH*	*T30- O4*′^*α*^	*28*.*52%*			
Cas1b				*Ile291-N*	*C28-O4*′^*α*^	*35*.*08%*
				*Ile291-N*	*C28-O2* ^*α*^	*34*.*68%*
				Gln287-NE2	T29-O2^α^	44.63%
				*Gln288-O*	*T30- O4*′^*α*^	*65*.*28%*
				*Gln287-NE2*	*T30- O4*′^*α*^	*45*.*24%*
Cas1a′	*Tyr165-OH*	*T28- O4*′^*β*^	*35*.*76%*	*Lys211-NZ*	*C28- O3*′^*β*^	*24*.*76%*
	*Arg138-NH1*	*T29- O4*′^*β*^	*57*.*56%*	***His208-NE2***	***T29-O1P*** ^***β***^	***91***.***48%***
	*Lys211-NZ*	*T29- O4*′^*β*^	*38*.*68%*	Arg138-O	T29-N3^β^	50.45%
	***His208-NE2***	***T29-O2P*** ^***β***^	***58***.***69%***	*Arg138-NH1*	*T29-O4* ^*β*^	*38*.*28%*
	Tyr165-N	T30-O2^β^	96.72%	*Arg146-NH2*	*T29-O2* ^*β*^	*21*.*36%*
	Tyr165-O	T30-N3^β^	95.54%	*Arg163-O*	*T30- O4*′^*β*^	*27*.*60%*
	*Tyr165-OH*	*T30- O4*′^*β*^	*78*.*64%*			
Cas1b′				*Ala290-N*	*C28-O2* ^*β*^	*40*.*72%*
				*Ile291-N*	*C28-O2* ^*β*^	*35*.*08%*

^※^HDO is the abbreviation of hydrogen bond occupancy.

^α^Nucleotide base belonging to α strand of DNA. ^β^Nucleotide base belonging to β strand of DNA.

Hydrogen bonds in non-italics and italics reflect the direct and water-mediated interactions, respectively. Bold denotes the crucial interactions involving site-specific cleavage of Cas1.

**Figure 8:**
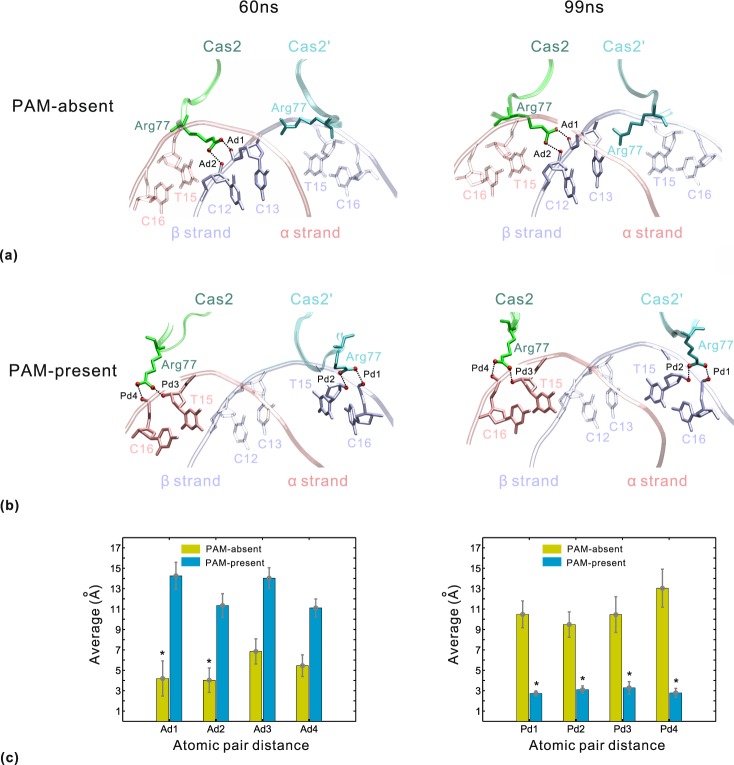
Protein-DNA interactions involving Arg77 in the PAM-absent (**a**) and PAM-present (**b**) systems, and the average distances of relevant atomic pairs (**c**). The Cas2/Cas2′ (green/cyan), DNA (α strand: pink; β strand: light blue) are depicted with ribbon and tube models, respectively. Atoms involved in protein-DNA interactions are shown in CPK. In the PAM-absent system (**a**), the side chain of Arg77 swings in the surface of DNA minor groove. In the PAM-present system (**b**), the side chain of Arg77 always orientates toward the DNA backbone and forms more arginine-DNA interactions. So the two systems have different atomic pair distance distributions (**c**). Ad3/Ad4 stands for the distance between Arg77 NH2/Arg77 NH1 in Cas2′ and C13 O3′/G12 O1P along α strand, which is not shown in (**a**) for clarity. Values represent mean ± S.D. (error bars) from the equilibrium trajectories. *Indicating the bond length of two bonded atoms labeled in (**a**,**b**).

The Cas2 dimer is responsible for contacting the duplex segment of protospacer. The hydrogen bonds at the interface of Cas2-DNA are shown in Table 2. In each system, the residues Asn10, Arg14, Arg16, Arg77 and Arg78 form contacts with the backbone of DNA. The protospacer sequence binds to Cas2 and Cas2′ mainly through arginine-DNA interactions between negatively charged phosphate backbone and positively charged protein surface^14,15^. The previous study^15^ revealed that Arg77 side chain undergoes a significant conformational change upon protospacer binding. Figure 8 shows a noteworthy difference of protein-DNA interaction involving Arg77 between the two systems. In the PAM-absent system (Fig. 8a), Arg77 adopts an energetically unfavorable conformation in which its side chain swings in the DNA minor groove surface. This high flexibility leads to the weakened hydrogen bond between Arg77 in Cas2 and the phosphate backbone, as well as the absence of protein-DNA contact of Arg77 in Cas2′. In the PAM-present system (Fig. 8b), Arg77 side chain flips about 90 degrees and this orientation allows more contacts with the phosphate backbone. So, Arg77 side chains in both Cas2 and Cas2′ form two high-occupancy hydrogen bonds with O3′ of T15 and O1P of C16, respectively (Table 2). We also measured the distances of relevant atomic pairs. Ad1/Ad2 stands for the distance between Arg77 NH2/Arg77 NH1 in Cas2 and C13 O3′/C12 O1P along β strand (Fig. 8a). Ad3/Ad4 stands for the distance between Arg77 NH2/Arg77 NH1 in Cas2′ and C13 O3′/G12 O1P along α strand (not shown in Fig. 8a for clarity). Pd1/Pd2 represents the distance between Arg77 NH2/Arg77 NH1 in Cas2′ and C16 O1P/T15 O3′ along β strand. Pd3/Pd4 represents the distance between Arg77 NH1/Arg77 NH2 in Cas2 and T15 O3′/C16 O1P along α strand (Fig. 8b). Obviously, the two systems have different atomic pair distance distributions (Fig. 8c). In the PAM-absent system, the average values of Ad1 and Ad2 have relatively high standard deviations while those of Ad3 and Ad4 are even greater than the distance cutoff for hydrogen bonds (3.5 Å). This distance distribution corresponds to a comparatively weak interaction between Arg77 and DNA. In the PAM-present system, the rearrangement of Arg77 side chain leads to a new distance distribution in which Pd1~Pd4 keep stable around 3 Å and support a robust hydrogen-bonding pattern. These results suggest that the proper orientation of Arg77 side chain is important for facilitating arginine-DNA interactions. Notably, these arginine-DNA interactions can enhance the deformability of DNA duplex by neutralizing the negative charge repulsions along the phosphate backbone^48^. The crystallographic study^15^ revealed an apparent bending of the Cas1-Cas2-bound duplex relative to the canonical B-form DNA by using the superposition method. Then, DNA deformations upon Cas1-Cas2 binding were investigated in the following section.

**Table 2:** The hydrogen bonds at the Cas2-DNA interface with occupancy over 20%.

Base pair	PAM-absent system			PAM-present system		
P_α_/P_β_^*^	Protein	DNA	HDO^※^	Protein	DNA	HDO
5/19	Cas2-Arg14-NH2	G5-O1P^α^	31.17%	Cas2-Arg14-NH2	G5-O1P^α^	24.20%
	Cas2-Arg14-NE	G5-O1P^α^	31.15%			
6/18				Cas2-Arg14-NH1	C6-O2P^α^	24.46%
8/16				**Cas2**′**-Arg77-NH2**	**C16-O1P** ^**β**^	**96**.**90%**
9/15	Cas2-Arg16-NH1	T15-O2P^β^	80.04%	Cas2-Arg16-NH1	T15-O2P^β^	69.57%
	Cas2-Arg16-NH2	T15-O2P^β^	73.43%	Cas2-Arg16-NH2	T15-O2P^β^	53.05%
	Cas2′-Arg78-NH1	T15-O1P^β^	62.13%	Cas2-Arg16-NH2	T15-O1P^β^	24.72%
	Cas2′-Arg78-NE	T15-O1P^β^	30.43%	**Cas2**′**-Arg77-NH1**	**T15-O3**′^**β**^	**90**.**00%**
	Cas2′-Arg78-NH2	T15-O1P^β^	25.17%	Cas2′-Arg78-NH2	T15-O1P^β^	72.51%
10/14	Cas2-Asn10-N	C14-O2P^β^	91.12%	Cas2-Asn10-N	C14-O1P^β^	22.74%
				Cas2-Asn10-ND2	C14-O2P^β^	22.68%
11/13	**Cas2-Arg77-NH2**	**C13-O1P** ^**β**^	**51**.**23%**	Cas2-Asn10-ND2	C13-O1P^β^	23.70%
	Cas2-Asn10-ND2	C13-O2P^β^	30.17%			
12/12	**Cas2-Arg77-NH1**	**C12-O3**′^**β**^	**36**.**97%**			
14/10	Cas2′-Arg16-NH2	C14-O2P^α^	61.31%	Cas2′-Asn10-ND2	C14-O2P^α^	28.25%
	Cas2′-Arg16-NH1	C14-O1P^α^	56.71%			
	Cas2′-Asn10-ND2	C14-O2P^α^	33.47%			
	Cas2′-Arg16-NH2	C14-O1P^α^	23.00%			
15/9	Cas2-Arg78-NH2	T15-O1P^α^	73.49%	Cas2′-Arg16-NH2	T15-O2P^α^	75.60%
	Cas2-Arg78-NE	T15-O1P^α^	66.63%	Cas2′-Arg16-NH1	T15-O2P^α^	61.19%
				**Cas2-Arg77-NH1**	**T15-O3**′^**α**^	**80**.**90%**
				Cas2-Arg78-NH2	T15-O1P^α^	85.36%
16/8				**Cas2-Arg77-NH2**	**C16-O1P** ^**α**^	**94**.**66%**
19/5	Cas2′-Arg14-NE	G5-O1P^β^	60.83%	Cas2′-Arg14-NE	G5-O1P^β^	24.26%
	Cas2′-Arg14-NH2	G5-O1P^β^	60.33%	Cas2′-Arg14-NH1	G5-O1P^β^	23.04%
20/4				Cas2′-Arg14-NH1	A4-O2P^β^	39.31%

^*^P_α_/P_β_ is the abbreviation of the nucleotide position along α/β strand. ^※^HDO is the abbreviation of hydrogen bond occupancy.

^α^Nucleotide base belonging to α strand of DNA. ^β^Nucleotide base belonging to β strand of DNA.

Hydrogen bonds in bold reflect the protein-DNA interactions involving Arg77.

### DNA deformations of duplex segments

**Figure 9:**
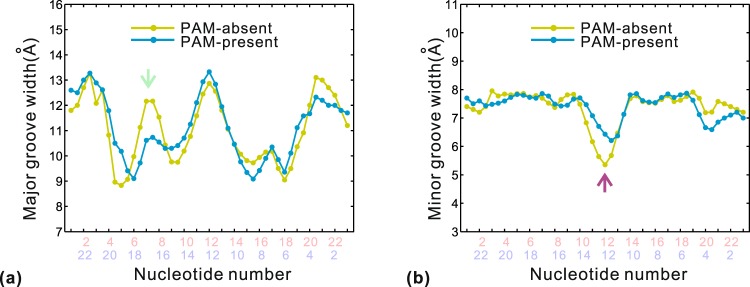
Average values of major (**a**) and minor (**b**) groove widths calculated from the equilibrium trajectories in the PAM-absent and PAM-present systems. The nucleotide numbers in pink and light blue correspond to α and β strands, respectively. The green and purple arrows mark the positions with the remarkable differences of major and minor groove widths between the two systems, respectively.

To analyze the DNA distortion, the structural parameters of the PAM-absent and PAM-present protospacer sequences were calculated with Curves program^43^ along the duplex segment (positions 1~23) from the equilibrium trajectories. The groove parameters are important structural features involving protein-DNA binding^32^. Figure 9 compares the average major and minor groove widths at bp level. The two DNA-bound systems have the similar variations in groove widths. Relative to the canonical B-form DNA (12 Å), the major groove of each system is narrowed to around 10 Å at positions 5~9 and 15~19 (Fig. 9a). The previous study described the remarkable ability of arginine to DNA compaction by neutralizing highly negatively charged DNA^49^. In our calculation, the reduced major groove widths mainly correspond to the arginine-mediated interactions from Arg14, Arg16, Arg77 and Arg78 (Table 2). The experimental data showed that no new spacer acquisition was observed for double mutants of Arg14Ala/Arg16Ala and Arg77Ala/Arg78Ala^15^. Then, these arginine-DNA interactions induce the major groove compression and stabilize the duplex segment. The exception is positions 7~8 in the PAM-absent sequence, showing an uncompressed major groove (Fig. 9a, marked by green arrow). Also, the minor groove widths of the two systems differ remarkably at position 12 (Fig. 9b, marked by purple arrow). These differences probably arise from the change of interactions involving Arg77 between the two systems (Fig. 8). In the PAM-absent system, only Arg77 in Cas2 contacts with the backbone of C12~C13 along β strand in the minor groove surface. It corresponds to the minor groove compression of ~1 Å relative to that of the PAM-present system (Fig. 9b). In the PAM-present system, residues Arg77 in both Cas2 and Cas2′ form the interaction with the phosphate groups of C16 along α and β strands, respectively. Correspondingly, the major groove nearby C16 is relatively more narrow than that of PAM-absent system in the two strands (Fig. 9a). The above groove-width analyses prove the important influence of arginine-DNA interactions on the Cas1-Cas2-protospacer binding.

**Figure 10:**
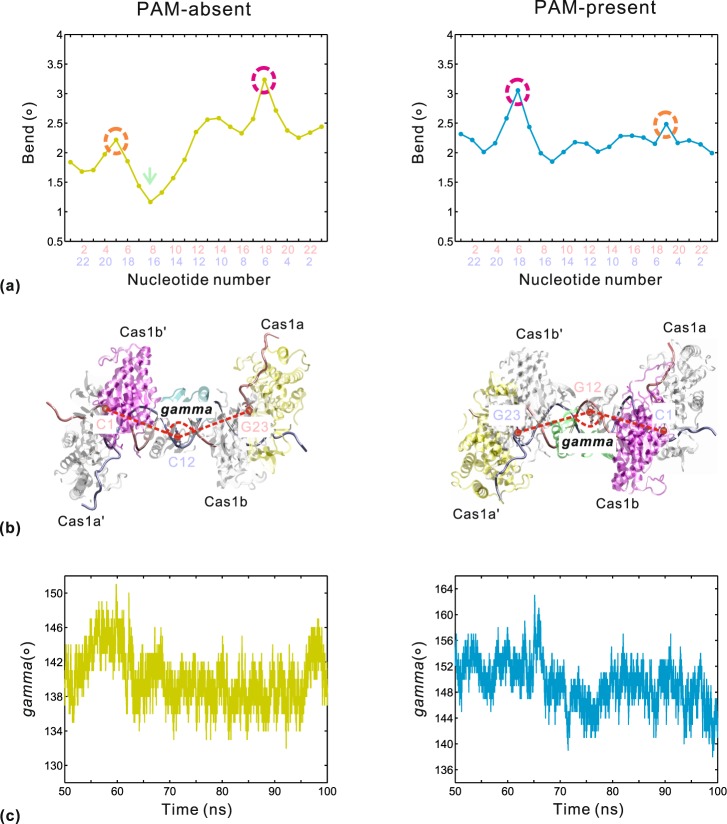
DNA bending degree calculated from the equilibrium trajectories in the PAM-absent (left) and PAM-present (right) systems. (**a**) Average values of the axis bend. The nucleotide numbers in pink and light blue correspond to α and β strands, respectively. Circles mark local maximums of bend. (**b**) The representation of *gamma*. It is defined by the geometric centers of the first, middle and last nucleotides along the duplex segment. (**c**) The changes of *gamma* versus simulation time.

It was documented that the charge-neutralization-induced DNA bending makes a significant contribution to the energetics of protein-DNA binding^48^. Figure 10a displays the mean bend angles at bp level, and the changes in bending between the two systems show some interesting points. Firstly, the bend angle values in each system exhibit two local maximums (Fig. 10a, marked by red and orange circles) but their positions are reverse distribution. We speculate that it might be associated with the open-close movements on the surfaces of Cas1a′-Cas2-Cas1b and Cas1a-Cas2′-Cas1b′, since the first slow motions of the two systems are in the opposite direction (Fig. 3b,d). Second, the two systems have completely different fluctuations of bend angle from position 8 to position 17. For this nucleotide segment, the PAM-present system shows a stable bend angle of about 2.1° (Fig. 10a right), which is induced by the charge neutralization of residues Arg77/Arg78/Arg16 in both Cas2 and Cas2′ (Table 2). In the PAM-absent system, this effect of the phosphate backbone neutralization is reduced due to the loss of arginine-DNA interaction from residue Arg77 in Cas2′. In this case, positions 8~17 show the bend angle fluctuating from 1.2° to 2.5° (Fig. 10a left). The lowest bend angle appears at position 8 (marked by green arrow), corresponding to the position of uncompressed major groove (Fig. 9a, marked by green arrow). In sum, the total bending of the PAM-absent system is relatively lower than that of the PAM-present system.

To confirm the above speculation about the reverse distribution of bend angles of the two systems, we further define an intramolecular angle *gamma* to provide an observation for the localized bending of DNA. The angle *gamma* is formed by the three geometric centers of the first, middle and last nucleotides along the duplex segment (Fig. 10b). As expected, the fluctuations of *gamma* (Fig. 10c) are similar to those of *alpha* versus simulation time (Fig. 4b). The correlation coefficients are 0.77 and 0.67 for the PAM-absent and PAM-present systems, respectively (Supplementary Fig. S2). The special relationship between *alpha* and *gamma* strongly suggests that the duplex bends in response to the open-close conformational change on the surfaces of Cas1a′-Cas2-Cas1b and Cas1a-Cas2′-Cas1b′. Thus, on the one hand, arginine-mediated interactions regulate DNA deformations at bp level; on the other hand, keeping a special DNA curvature is the intrinsic nature of Ca1-Cas2-protospacer. Taken together, we propose that the deformation of protospacer DNA is important not only for binding to Cas1-Cas2 protein, but also for a cooperative binding with Cas1-Cas2 for capturing the potential target DNA. This cooperativity between Cas1-Cas2 and protospacer will enable the high-efficient formation of the CRISPR locus integration complex.

## Conclusions

In this study, MD simulations were performed to investigate the binding mechanism of Cas1-Cas2 and protospacer DNA. The comparative analyses of MD trajectories indicated that the protospacer binding improves the stability of Cas1-Cas2, in particular when the PAM-complementary sequence is present in the 3′ flanking regions of protospacer. The PCA analyses revealed that the two protospacer DNA-bound (PAM-absent and PAM-present) systems have highly similar slow modes, including the open-close movements on the surfaces of Cas1a′-Cas2-Cas1b and Cas1a-Cas2′-Cas1b′ from PC1, as well as the rotation motions in reverse direction between the two Cas1 dimers from PC2. The open-close movements of PC1 and the rotation motions of PC2 cause the changes of intramolecular angles *alpha* and *beta*, respectively. Next, the intramolecular angle analyses showed that the rotation motions correspond to the requirement of structural rearrangement of Cas1-Cas2 upon protospacer binding, and the open-close motions are linked to the binding between Cas1-Cas2-protospacer and the potential target DNA. Despite undergoing the similar conformational change, the PAM-present system achieves low-energy conformations more efficiently relative to the PAM-absent system.

To explain the change of DNA-binding efficiency, we analyzed the interfacial interactions by using the MM/PBSA method in combination with the hydrogen bond calculation. Relative to the PAM-absent system, the PAM-complementary sequence in the PAM-present system forms more specific contacts to increase the cleavage activity of Cas1 protein. Meanwhile, the proper conformation of Arg77 side chain in Cas2 protein also facilitates arginine-DNA interactions with the phosphate backbone. These crucial interactions help the PAM-complementary-containing protospacer to bind more effectively with Cas1-Cas2.

In the last part, the deformability of duplex DNA upon Cas1-Cas2 binding was investigated. On the one hand, the variations of major groove width and bend angle at bp level are regulated by the arginine-DNA interactions between arginine residues (Arg14, Arg16, Arg77 and Arg78) and the phosphate backbone. On the other hand, the localized bending of duplex has an apparent correlation with the open-close conformational change of Cas1-Cas2 from PC1. Finally, we proposed that the cooperative behaviour between Cas1-Cas2 and protospacer is the intrinsic requirement of the target DNA capture, which will favor the formation of the CRISPR locus integration complex. This study gives the dynamics and atomic level information for Cas1-Cas2-protospacer binding, and provides some new insights into the spacer acquisition machinery in CRISPR-Cas systems.

## Supplementary information

Supplementary Information

## Data Availability

The data generated and analyzed in this study are available from the corresponding author on reasonable request.

## References

[1] Barrangou R (2007). CRISPR provides acquired resistance against viruses in prokaryotes. Science.

[2] Brouns SJJ (2008). Small CRISPR RNAs guide antiviral defense in prokaryotes. Science.

[3] Barrangou R, Marraffini LA (2014). CRISPR-Cas Systems: Prokaryotes Upgrade to Adaptive Immunity. Mol. Cell.

[4] van der Oost J, Jore MM, Westra ER, Lundgren M, Brouns SJJ (2009). CRISPR-based adaptive and heritable immunity in prokaryotes. Trends Biochem. Sci..

[5] Garneau JE (2010). The CRISPR/Cas bacterial immune system cleaves bacteriophage and plasmid DNA. Nature.

[6] Nunez JK (2014). Cas1-Cas2 complex formation mediates spacer acquisition during CRISPR-Cas adaptive immunity. Nat. Struct. Mol. Biol..

[7] Jackson SA (2017). CRISPR-Cas: Adapting to change. Science.

[8] Deveau H (2008). Phage response to CRISPR-Encoded resistance in Streptococcus thermophilus. J. Bacteriol..

[9] Mojica FJM, Diez-Villasenor C, Garcia-Martinez J, Almendros C (2009). Short motif sequences determine the targets of the prokaryotic CRISPR defence system. Microbiology-(UK).

[10] Semenova E (2011). Interference by clustered regularly interspaced short palindromic repeat (CRISPR) RNA is governed by a seed sequence. Proc. Natl. Acad. Sci. USA.

[11] Swarts DC, Mosterd C, van Passel MWJ, Brouns SJJ (2012). CRISPR Interference Directs Strand Specific Spacer Acquisition. PLoS One.

[12] Makarova KS (2011). Evolution and classification of the CRISPR-Cas systems. Nat. Rev. Microbiol..

[13] Yosef I, Goren MG, Qimron U (2012). Proteins and DNA elements essential for the CRISPR adaptation process in Escherichia coli. Nucleic Acids Res..

[14] Nunez JK, Harrington LB, Kranzusch PJ, Engelman AN, Doudna JA (2015). Foreign DNA capture during CRISPR-Cas adaptive immunity. Nature.

[15] Wang J (2015). Structural and Mechanistic Basis of PAM-Dependent Spacer Acquisition in CRISPR-Cas Systems. Cell.

[16] Wright AV (2017). Structures of the CRISPR genome integration complex. Science.

[17] Xiao YB, Ng S, Nam KH, Ke AL (2017). How type II CRISPR-Cas establish immunity through Cas1-Cas2-mediated spacer integration. Nature.

[18] Haft DH, Selengut J, Mongodin EF, Nelson KE (2005). A guild of 45 CRISPR-associated (Cas) protein families and multiple CRISPR/Cas subtypes exist in prokaryotic genomes. PLoS Comput. Biol..

[19] Martynov A, Severinov K, Ispolatov I (2017). Optimal number of spacers in CRISPR arrays. PLoS Comput. Biol..

[20] Pinello L (2016). Analyzing CRISPR genome-editing experiments with CRISPResso. Nat. Biotechnol..

[21] Zheng WJ (2017). Probing the structural dynamics of the CRISPR-Cas9 RNA-guided DNA-cleavage system by coarse-grained modeling. Proteins: Struct. Funct. Bioinform..

[22] Zeng Y (2018). The initiation, propagation and dynamics of CRISPR-SpyCas9 R-loop complex. Nucleic Acids Res..

[23] Xu, X., Duan, D. & Chen, S. J. CRISPR-Cas9 cleavage efficiency correlates strongly with target-sgRNA folding stability: from physical mechanism to off-target assessment. *Sci*. *Rep*. **7** (2017). 10.1038/s41598-017-00180-1 PMC5427927 28273945

[24] Datsenko KA (2012). Molecular memory of prior infections activates the CRISPR/Cas adaptive bacterial immunity system. Nat. Commun..

[25] Phillips JC (2005). Scalable molecular dynamics with NAMD. J. Comput. Chem..

[26] Vanommeslaeghe K (2010). CHARMM General Force Field: A Force Field for Drug-Like Molecules Compatible with the CHARMM All-Atom Additive Biological Force Fields. J. Comput. Chem..

[27] Humphrey, W., Dalke, A. & Schulten, K. VMD: visual molecular dynamics. *J*. *Mol*. *Graphics* **14****(****1**), 33–38, 27–38 (1996). 10.1016/0263-7855(96)00018-5 8744570

[28] Hatano T, Sasa S (2001). Steady-state thermodynamics of Langevin systems. Phys. Rev. Lett..

[29] Ryckaert JP, Ciccotti G, Berendsen HJC (1997). Numerical integration of the cartesian equations of motion of a system with constraints: molecular dynamics of n-alkanes. J. Comput. Phys..

[30] Darden T, York D, Pedersen L (1993). Particle mesh Ewald: an N.Log(N) method for Ewald sums in large systems. J. Chem. Phys..

[31] Wan H, Hu JP, Tian XH, Chang S (2013). Molecular dynamics simulations of wild type and mutants of human complement receptor 2 complexed with C3d. Phys. Chem. Chem. Phys..

[32] Wan H, Chang S, Hu JP, Tian YX, Tian XH (2015). Molecular Dynamics Simulations of Ternary Complexes: Comparisons of LEAFY Protein Binding to Different DNA Motifs. J. Chem Inf. Model..

[33] Nguyen PH, Li MS, Derreumaux P (2011). Effects of all-atom force fields on amyloid oligomerization: replica exchange molecular dynamics simulations of the A beta(16–22) dimer and trimer. Phys. Chem. Chem. Phys..

[34] David CC, Jacobs DJ (2014). Principal Component Analysis: A Method for Determining the Essential Dynamics of Proteins. Methods Mol. Biol..

[35] Hess B (2002). Convergence of sampling in protein simulations. Phys. Rev. E.

[36] Van der Spoel D (2005). GROMACS: Fast, flexible, and free. J. Comput. Chem..

[37] Onuchic JN, Luthey-Schulten Z, Wolynes PG (1997). Theory of protein folding: the energy landscape perspective. Annu. Rev. Phys. Chem..

[38] Sun HY (2014). Assessing the performance of MM/PBSA and MM/GBSA methods. 5. Improved docking performance using high solute dielectric constant MM/GBSA and MM/PBSA rescoring. Phys. Chem. Chem. Phys..

[39] Sun H, Li Y, Tian S, Xu L, Hou T (2014). Assessing the performance of MM/PBSA and MM/GBSA methods. 4. Accuracies of MM/PBSA and MM/GBSA methodologies evaluated by various simulation protocols using PDBbind data set. Phys. Chem. Chem. Phys..

[40] Chen F (2016). Assessing the performance of the MM/PBSA and MM/GBSA methods. 6. Capability to predict protein-protein binding free energies and re-rank binding poses generated by protein-protein docking. Phys. Chem. Chem. Phys..

[41] Sun H (2018). Assessing the performance of MM/PBSA and MM/GBSA methods. 7. Entropy effects on the performance of end-point binding free energy calculation approaches. Phys. Chem. Chem. Phys..

[42] Kumari R, Kumar R, Lynn A (2014). g_mmpbsa-A GROMACS Tool for High-Throughput MM-PBSA Calculations. J. Chem Inf. Model..

[43] Lavery R, Moakher M, Maddocks JH, Petkeviciute D, Zakrzewska K (2009). Conformational analysis of nucleic acids revisited: Curves. Nucleic Acids Res..

[44] Maisuradze GG, Liwo A, Scheraga HA (2009). Principal Component Analysis for Protein Folding Dynamics. J. Mol. Biol..

[45] Maisuradze GG, Leitner DM (2007). Free energy landscape of a biomolecule in dihedral principal component space: Sampling convergence and correspondence between structures and minima. Proteins: Struct. Funct. Bioinform..

[46] Chang S (2016). Exploring the molecular basis of RNA recognition by the dimeric RNA-binding protein via molecular simulation methods. RNA Biol..

[47] Yam SC, Zain SM, Lee VS, Chew KH (2018). Correlation between polar surface area and bioferroelectricity in DNA and RNA nucleobases. Eur. Phys. J. E.

[48] Okonogi TM, Alley SC, Harwood EA, Hopkins PB, Robinson BH (2002). Phosphate backbone neutralization increases duplex DNA flexibility: A model for protein binding. Proc. Natl. Acad. Sci. USA.

[49] DeRouchey J, Hoover B, Rau DC (2013). A Comparison of DNA Compaction by Arginine and Lysine Peptides: A Physical Basis for Arginine Rich Protarnines. Biochemistry.

