# Supplementary material for: Probing the Behaviour of Cas1-Cas2 upon Protospacer Binding in CRISPR-Cas Systems using Molecular Dynamics Simulations
Source: Sci Rep. 2019 Feb 28;9:3188. doi: 10.1038/s41598-019-39616-1 (PMC6395717; doi:10.1038/s41598-019-39616-1)
Supplement: Supplementary file 1 — Supplementary Information [file 41598_2019_39616_MOESM1_ESM.pdf]

Supplementary Information:

Probing the Behaviour of Cas1-Cas2 upon Protospacer Binding in CRISPR-Cas Systems using Molecular Dynamics Simulations

Hua Wan [1], Jianming Li [1], Shan Chang [2], Shuoxin Lin [3], Yuanxin Tian [4], Xuhong Tian [1], Meihua Wang [1*], Jianping Hu [5*]

[1] *College of Mathematics and Informatics, South China Agricultural University, Guangzhou 510642, China.*

[2] *Institute of Bioinformatics and Medical Engineering, School of Electrical and Information Engineering, Jiangsu University of Technology, Changzhou 213001, China*

[3] *Department of Electrical and Computer Engineering, James Clark School of Engineering, University of Maryland, College Park, MD 20742, USA*

[4] *School of Pharmaceutical Sciences, Southern Medical University, Guangzhou 510515, China*

[5] *College of Pharmacy and Biological Engineering, Sichuan Industrial Institute of Antibiotics, Key Laboratory of Medicinal and Edible Plants Resources Development of Sichuan Education Department, Antibiotics Research and Re-evaluation Key Laboratory of Sichuan Province, Chengdu University, Chengdu 610106, China. E-mail: hjpcdu@163.com*

Figures:

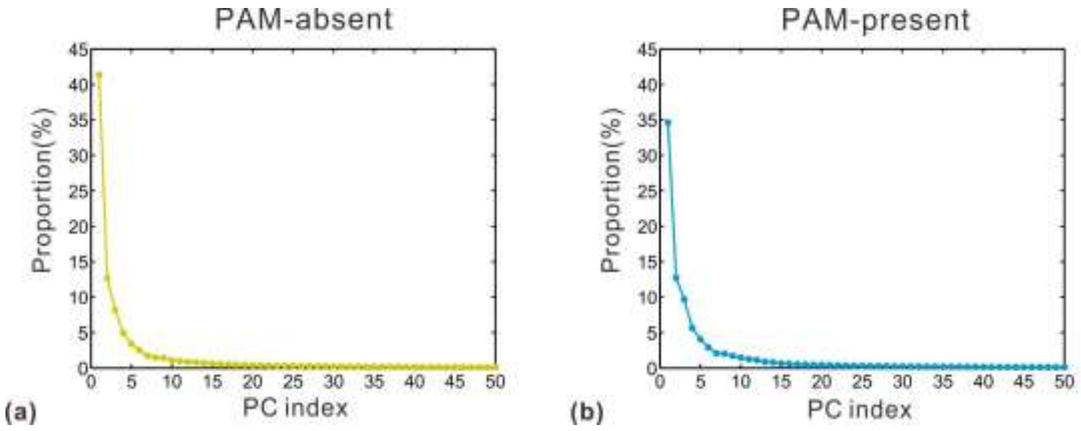

Figure S1. Eigenvalue contribution of the first 50 PCs to system's variance. (a) The PAM-absent system. (b) The PAM-present system.

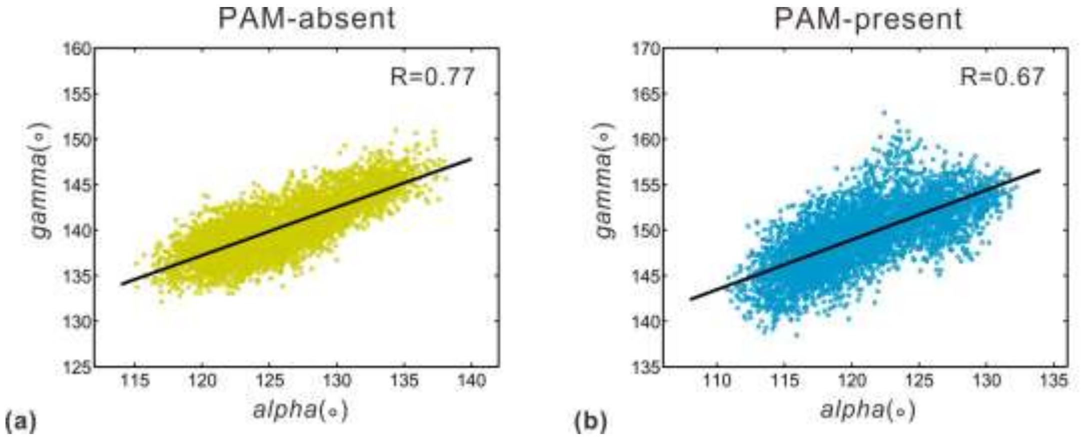

Figure S2. Correlation between the two intramolecular angles of *alpha* and *gamma*. (a) The PAM-absent system. (b) The PAM-present system.

Tables:

Table S1. Cosine contents of the first eight principal components for the PAM-absent and PAM-present systems.

Systems	PC1	PC2	PC3	PC4	PC5	PC6	PC7	PC8
**PAM-absent**	0.3274	0.0267	0.0291	0.1218	0.0749	0.0664	0.0057	0.0084
**PAM-present**	0.3850	0.3157	0.3716	0.2808	0.0070	0.1453	0.0038	0.0279

Table S2. Components and standard errors of binding energies between the binding pocket of Cas1 protein and the nucleotide segment at positions 28~30.

Interfaces		ΔE_{vdw}*	ΔE_{elec}	ΔG_{polar}	$\Delta G_{nonpolar}$	$\Delta G_{binding}$
**I1**#	**PAM-absent**	-184.69±19.67	-925.25±101.66	738.53±99.96	-25.83±1.07	-397.24±44.07
	**PAM-present**	-244.78±19.48	-967.00±38.96	803.71±35.74	-32.67±1.04	-440.74±27.91
**I2**$	**PAM-absent**	-189.75±20.47	-936.61±54.22	735.19±45.64	-27.38±1.08	-418.54±33.84
	**PAM-present**	-231.83±16.65	-1016.29±65.81	811.23±64.15	-30.55±1.45	-467.44±23.70

*ΔE_{vdw}, ΔE_{elec}, ΔG_{polar}, and $\Delta G_{nonpolar}$ are binding energy components of van der Waals, electrostatic, polar and nonpolar solvation energies, respectively. $\Delta G_{binding}$ is the total binding energy. #I1 stands for the protein-DNA interface between the binding pocket of Cas1a-Cas1b and α strand of DNA, and $I2 stands for the protein-DNA interface between the binding pocket of Cas1a'-Cas1b' and β strand of DNA. The energies are in kilojoules per mole.
